# Influences of pulsed electric field parameters on cell electroporation and electrofusion events: Comprehensive understanding by experiments and molecular dynamics simulations

**DOI:** 10.1371/journal.pone.0306945

**Published:** 2025-01-22

**Authors:** Sujun Qu, Qiang Ke, Xinhao Li, Lin Yu, Shuheng Huang

**Affiliations:** 1 Department of Pharmacy, Jingmen Central Hospital, Jingmen Central Hospital Affiliated to Jingchu University of Technology, Jingmen, Hubei, China; 2 Nanjing Research Institute of Electronics Technology, Nanjing, China; 3 State Key Laboratory of Power Transmission Equipment & System Security and New Technology, School of Electrical Engineering, Chongqing University, Chongqing, China; 4 Institute of Translational Medicine, Medical College, Yangzhou University, Yangzhou, Jiangsu, China; 5 Key Laboratory of Tropical Biological Resources of Ministry of Education, School of Pharmaceutical Sciences, Hainan University, Haikou, China; National Research Council: Consiglio Nazionale delle Ricerche, ITALY

## Abstract

Electroporation and electrofusion are efficient methods, which have been widely used in different areas of biotechnology and medicine. Pulse strength and width, as an external condition, play an important role in the process of these methods. However, comparatively little work has been done to explore the effects of pulsed electric field parameters on electroporation and electrofusion. Herein, influences of pulse strength and width on the electroporation and electrofusion of phospholipid bilayers were systematically investigated by using experiments combined with molecular dynamics simulations. Experimental results and machine learning-based regression analysis showed that the number of pores is mainly determined by pulse strength, while the sizes of pores were enlarged by increasing the pulse widths. In addition, the formation of large-size pores is the most crucial factor that affects the fusion rate of myeloma cells. The same trend has taken place on coarse-grained and all-atom MD simulations. The result suggested that electroporation events occur only in an electric field exceeding the strength of threshold, and the unbalanced degree of electric potential between two membranes leads to pores formation during the process of electroporation. Generally, this work provides a comprehensive understanding of how pulse strength and width govern the poration event of bilayer lipid membranes, as well as guidance on the experimental design of electrofusion.

## Introduction

Electroporation, also known as electropermeabilization, occurs when the cell membrane suffers intense amplitude to temporarily increased permeability by exposure to short electric pulses [1,2]. Morphological studies indicated that when two or more cells contact each other under pulsed electric field (PEF), their plasmalemmas can generate nanopores, which induce the rearrangement of phospholipid molecules and trigger the process of cell fusion [3,4]. In the past few decades, electroporation-induced electrofusion has become a well-established technique and is widely used in different areas of biotechnology and medicine, such as artificial transformation, cell hybridization, and cancer treatments [5–8].

Recently, a number of methods with nanosecond [9–11], bipolar [12,13], microsecond and sub-microsecond (nanosecond) pulses [14–16] have been proposed to improve the cell electroporation or fusion efficiency. Rems et al. [14] investigated the possibility of cell fusion induced by nanosecond pulses. The results of B16-F1 cell fusion experiments showed that the nanosecond pulses can obtain comparable fusion yields to microsecond pulses. Our previous work [15] proposed to combine nanosecond and microsecond pulses to fuse cells with different sizes. The results showed that compared to microsecond pulses, the combined pulses can induce a larger pore radius and remarkably improve cell fusion efficiencies. In addition, bipolar pulses with microsecond pulse widths were further performed on the electrofusion of mouse myeloma and lymphocytes cells with satisfactory results [13,17].

Undoubtedly, the parameters of PEF, such as pulse strengths and widths, play pivotal roles in determining the efficacy of cell electroporation and electrofusion techniques. It is essential to finely tune these PEF conditions to optimize outcomes for specific cell sizes or types. To date, extensive research has been conducted on how PEFs influence electroporation and electrofusion processes. For instance, Weaver et al. [18] have provided a comprehensive overview of electroporation applications, suggesting optimal ranges for PEF strength and duration to achieve desired effects. However, much of the existing research has primarily focused on examining aspects such as membrane permeabilization and pore formation, as well as the direct consequences of these events. How PEF parameters affect cell membrane permeability and fusion and its underlying molecular mechanisms continue to be exciting research puzzles.

In the process of electroporation, pores are too small to be observed due to their nanometer-level sizes. With the growth of computer technology, the molecular dynamics (MD) technique has shown tremendous promise and provides a significant insight into the process of electroporation. Accumulated evidence suggested that the rate of pore formation of phospholipid bilayers increases remarkably when exposure to a sufficiently strong electric field [19–22]. Most recently, MD simulation has also been successfully utilized to elucidate the experimental findings of the sustainable permeability of cell membranes induced by lipid peroxidation [23].

In this work, *in vitro* experiments combined with molecular dynamics simulations were performed to explore the influences of PEF parameters on electroporation and electrofusion of phospholipid bilayer membranes. Experimental results and machine learning-based regression analysis showed that the number of pores increases with pulse strength, and the sizes of pores were enlarged by the pulse width. Besides, the formation of large-size pores is the most crucial factor affecting myeloma cells’ fusion rate. The detailed mechanism of electroporation is further revealed by both coarse-grained (CG) and all-atom MD simulations. The results showed that the potential differences between lipid membranes mainly cause the pore creation of electroporation, and the unbalanced degree of electric potential leads to a different pore size during electroporation. Collectively, our findings provide a comprehensive understanding of how PEF conditions govern the poration event of bilayer lipid membranes, as well as guidance on the experimental design of electrofusion.

## Materials and methods

### Buffer, cell culture and experimental instrument

In staining experiments, buffer was constituted of Mg^2+^ (0.01 mmol/L), Ca^2+^ (0.01 mmol/L), bovine serum albumin (1 mg/mL) and D-Mannitol (120 mmol/L). Mouse SP2/0 myeloma cells were lysed in the buffer as cell suspension for further electrical stimulation. The mouse SP2/0 myeloma cells were first retrieved from liquid nitrogen. The cells were cultured and passed (sub-cultured) under a humidified atmosphere of 5% CO2 at 37 °C in RPMI 1640 with 10% fetal bovine serum. Lymphocytes were harvested from spleens of special pathogen-free (SPF) Kunming mice. Due to the low survival rate of lymphocytes, all experiments were carried out within six hours of cell isolation.

Herein, cell electrode groove and optical microscopy were employed for cell electroporation and electrofusion. The single-polarity pulse was used in the simulation. S1 Fig shows the schematic diagram of the homemade instrument. The cathodes of the pulse generator, power amplifier (TEGAM Corporation) and oscilloscope (Tektronix Corporation) were connected (black line). S1 and S2 were used to switch the sine waves and pulse waves, respectively. First, S1 was on, and S2 was off. Sine waves were applied to the cells to form pearl chains. Second, S2 was on, and S1 was off. Electric pulses were applied to the cell electrode groove.

### Cell culture and electroporation protocols

Herein, YO-PRO-1 (YP) and propidium iodide (PI) cell staining assays were employed to detect the formation of nanopores in the electroporation. YP and PI are one of the most efficient nucleic acid stains that can dissociate into propidium cations in water and bind to DNA by intercalating between the bases [24]. The maximum diameter of a propidium cation is about 1.5 nm, which is substantially larger than the YO-PRO-1 molecule (0.5–1.0 nm) [25]. Thus, the YP is mainly used to determine the cell pores with small sizes, while the PI can detect the formations of those pores with large sizes.

Cell density was examined with a microscope and adjusted to 1×10^6^ cells/mL by counting on a hemacytometer. A 0.5 ml aliquot was removed, and cells were washed and resuspended with a 5 ml balanced saline solution using a cytocentrifuge (1000 rpm/min, 3 min). After that, the washed cells were resuspended with another 0.5 mL cell buffer containing either YP or PI fluorescent probes to obtain cell suspension. They were then injected into the cell electrode groove. Finally, impulse voltage pulses were applied at the electrode. The images were taken by an inverted fluorescent microscope (DMi8, Leica Microsystems) after one hundred seconds of pulses to calculate the percentage of the total fluorescence of the cell.

### Electrofusion protocols

Cell density was examined with a microscope and adjusted to 1×10^6^ cells/mL by counting on a hemacytometer. A 0.5 ml aliquot was cultured with 0.5 ml Hoechst 33342 at 37°C for 20 min, and then suspended twice with 5 ml balanced saline solution using a cytocentrifuge (1000 rpm/min, 3 min) to remove excess dyes. After that, the cells were centrifuged again and then resuspended in an extracellular solution (5 mL).

For easy visualization of positively stained cells, Hoechst dye 33342 was used for nucleus staining before cell electrofusion. In the process of electrofusion, pulsed electric fields with a high-frequency sinusoidal field (100 V/cm, 1MHz) in the duration of 20s were employed in the cell suspension. Subsequently, a buffer containing PI fluorophores (1 mg/mL) was added to cells after 20 min of treatment. In this work, mouse SP2/0 myeloma cells were exposed to seven gradient pulse strengths (i.e., 0, 0.4, 0.8, 1.2, 1.6, 2.0, or 2.4 kV/cm) and four different widths (200 ns, 1000 ns, 10 μs or 40 μs). Five visual fields were chosen randomly in one culture dish by fluorescent microscope to detect the staining with the DNA binding dye Hoechst 33342. Herein, bright field and fluorescence images were collected to examine the cell electrofusion, where two or more cell nucleus were detected were judged as positive.

### Partial least squares modeling

Partial least squares (PLS) regression is a comprehensive machine learning method combined with principal components analysis (PCA) and multiple linear regression (MLR), which has been widely used in data processing and statistics [26,27]. PLS can establish the new predictor variables (i.e., principal components) from original predictor variables and then construct the linear combinations to fit the regression model [28,29]. In the process of PLS, X and Y are firstly bilinearly decomposed and then projected into a new principal components space:

$$X={TP}^{T}+E$$ (1)

$$Y={UC}^{T}+G$$ (2)

Where *T* and *P* represent the scores and loading matrices of X; *E* is the residual matrices. *U* and *C* represent the scores and weight matrices of Y, and G is the residual matrices. PLS aims to construct reliable relationships between X and Y, as follows:

$$Y={TC}^{T}+F$$ (3)

Where *F* is the residual matrix of Y.

### Molecular dynamics simulation protocols

#### Systems preparation

In this work, CG and all-atom models were used to explore the electroporation event of the cell membrane. The membrane models are equilibrated fully hydrated 1,2-dipalmitoyl-sn-glycero-3-phosphocholine (DPPC) bilayers. At physiological temperature (i.e., 310 K), DPPC bilayers are in the biologically relevant liquid crystal Lα phase. S2 Fig shows the molecular structure of DPPC in CG and all-atom models. Firstly, an extensive CG system with an assembly of 2858 DPPC bilayers was constructed to investigate the size effect. Each coarsely granulated DPPC molecule contained 12 CG particles, including eight hydrophobic particles (C1-C4), two head-charged particles (negatively charged PO4 and positively charged NC3), and two glycerol CG particles (GL1 and GL2).

Although the CG model is efficient in large-scale MD simulations, the loss in atomic details limits quantitative studies of many complex biological processes. Thus, another small all-atom system was constructed to explore the pore creation and biophysical properties at the atomic level. Each all-atom system contains 318 DPPC molecules solvated by a TIP3P water box with a water thickness of 30 Å. Both CG and all-atom systems were modeled and refined by charm GUI. To eliminate the bad contacts in the initial geometries, CG and all-atom systems were first optimized by 5000 steps of the steepest descent followed by 5000 steps of conjugate gradient energy minimization. Then, the energy-minimized bilayer lipid membranes structures were used for further MD production.

#### Molecular dynamics production

MD simulations with periodic boundary conditions [30,31] were carried out by the GROMACS package (versions 2020.5) [32,33]. CHARMM36 [34] and Martini 2.2 forcefield [35] were used in the all-atom and CG systems MD simulations, respectively. The integration time step was set to 20 fs for the CG systems and 2 fs for the all-atom system. The Parrinello-Rahman barostat was used for MD production with a reference pressure of 1 bar, of which the time constant and isothermal compressibility was set to 4 ps and 3×10^−4^ bar^-1^, respectively. Moreover, Verlet cutoff scheme was used for MD simulations, where the electrostatic cutoff and van der Waals cutoff were set as 1.1nm. The Coulomb interactions were screened by a relative permittivity constant of 15. The particle mesh Ewald (PME) method was used to calculate the long-range electrostatic interactions [36] and the SHAKE algorithm [37] was used to constrain the covalent bonds with H atoms.

In MD production, each system was firstly gradually heated from 0 to 310 K within 5000 ps in the NVT ensemble, where the heavy atoms of DPPC were harmonically constrained by a force of 100 kcal/mol·Å^2^. Then, 30 ns equilibrium MD simulations were performed in the NPT ensemble (310 K, 1 atm) without any constraint. Finally, a serial of gradient pulse strength was employed in 10 ns MD production to explore the electroporation process. Membrane tension develops within lipid bilayers and maintains cell shape and size. The change of cell membrane tensions is a complicated process that MD simulations are not able to simulate with accuracy. Our pre-experiment showed that the bilayers was completely destroyed when a high electric field was applied (S2C Fig), which affected the accuracy of research. For avoiding the bilayers burst under a high pulse strength, in the all-atom MD simulation, the Cα atoms of glycerol located in the outer layer (R > 40 Å) were harmonically constrained by the force of 50 kcal/mol·Å^2^ (S2D Fig).

#### Electrostatic properties analysis

The electrostatic properties of lipid membranes play a crucial role in cellular physiology as they are intricately linked to the membrane potential. Here, atomic-scale molecular dynamics simulations were employed to compute the electrostatic potential across lipid bilayers. These simulations offer a detailed view of the spatial distribution and temporal fluctuations of the electrostatic field, enabling a deeper understanding of how changes in electric field conditions affect membrane functionality. The electrostatic potential across the lipid membrane systems was derived from MD simulations by Poisson’s equation [38,39]. The electrostatic potential (∅(*z*)) is firstly calculated summing the charges per slice along *z*, and then integrating twice of the molecular charge density distributions, as follows:

$$\emptyset (z)=\emptyset (z)-\emptyset_{0}=\frac{-1}{\varepsilon_{0}}\iint_{0}^{z}\rho (z”)dz”dz\prime$$ (4)

Where, ∅(*z*) and ∅_0_ represent the electrostatic potential calculated in on *z* and starting position; *ρ*(*z*) and *ε*_0_ represent the molecular charge density distributions and the relative dielectric constant. Herein, ∅_0_ and *ε*_0_ are set to 0 and 1, respectively.

## Results

### Effects of PEFs on cell permeabilization by electroporation

**Fig 1:**
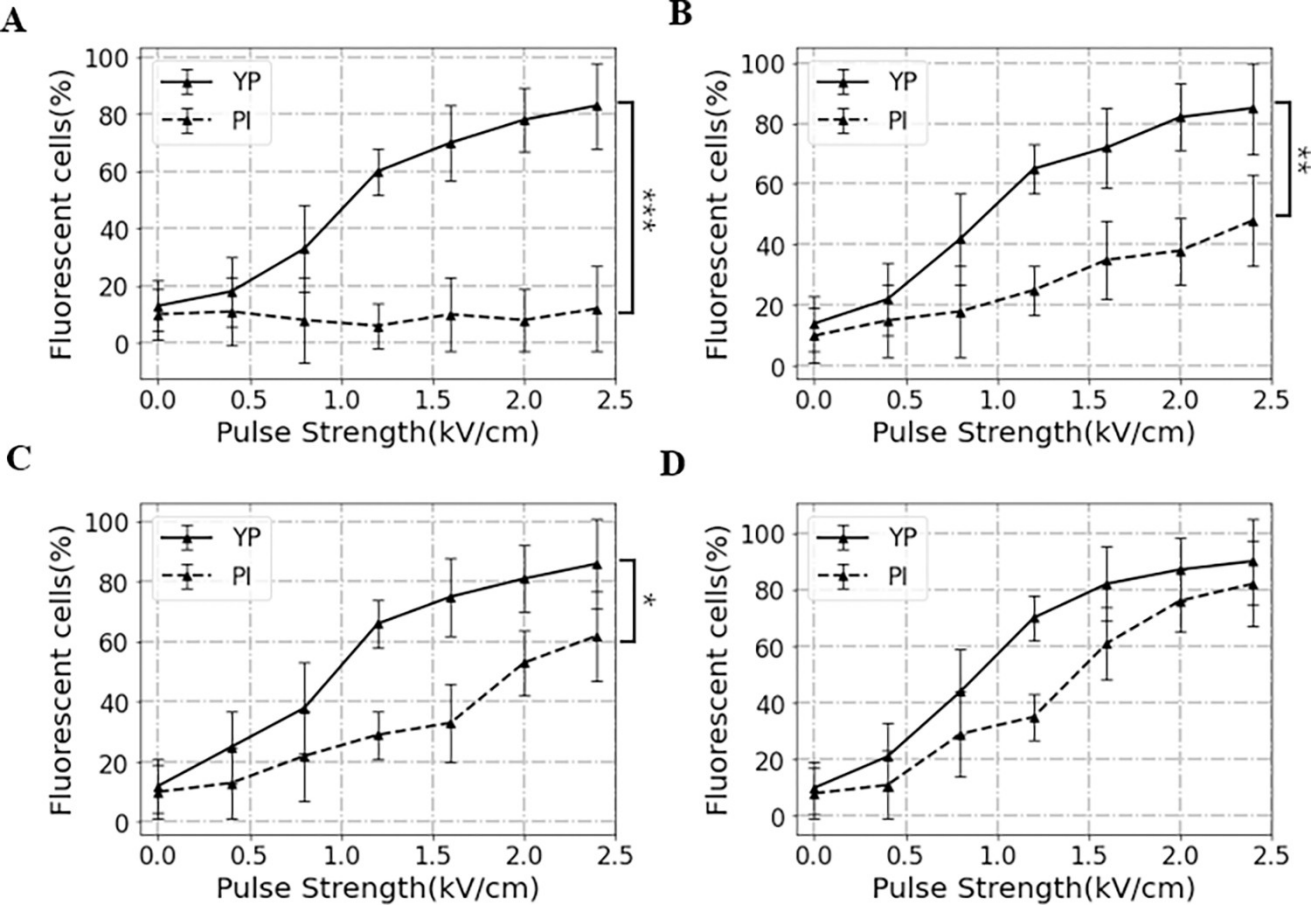
Percentage of fluorescent cells as determined by fluorescent microscopy on the pulse width of (A) 200ns, (B) 1000ns, (C) 10μs, and (D) 40μs. Data are means from five individual experiments; error bars show standard deviation. Asterisks *(P, 0.05), ** (P, 0.01) and *** (P, 0.001) mark significant difference between YP and PI groups.

YP and PI fluorescent dye-based cell staining techniques were employed to investigate the influences of pulse conditions on cell permeabilization induced by electroporation. In this work, mouse SP2/0 myeloma cells were first exposed to seven gradient pulse strengths (i.e., 0, 0.4, 0.8, 1.2, 1.6, 2.0, or 2.4 kV/cm) and four different widths (200 ns, 1000 ns, 10 μs or 40 μs). For the electrofusion protocols, a squared sinusoidal-shaped waveform was utilized (S1 Fig). As shown in Fig 1, in both YP and PI assays, it can be observed that the electroporation yield increased with the increase of pulse width and strength. Notably, the percentage of YP-positive cells detected was higher than 80% in all pulse width conditions when adopting a high-level pulse strength (2.4 kV/cm), indicating that the high-level pulse strength is a crucial factor that affects pores formation.

Compared with YP, PI seems to demonstrate lower sensitivities in detecting electroporation of mouse myeloma cells since its size is larger than YP. Although the pores that emerge after a pulse width of nanosecond level are usually too small to allow PI to pass (Fig 1A), PI can be used to detect those “nanopores” with larger sizes to some degree [25]. Similarly, the results of PI assay showed that the number of large-size “nanopores” demonstrates a dependence on pulse strength (Fig 1B–1D). Besides, it can be also seen that yield of YP-positive cells increased remarkably with increasing the pulse duration. The number of PI-positive cells detected was 62 ± 8% and 82 ± 3% of all cells after PEF pulse of 10 μs and 40 μs (2.4 kV/cm), respectively. The results implied that compared to low- or middle-level pulse width, a longer pulse duration (such as 40 μs) contributes to enlarging the cell pores. Collectively, electroporation yield increased when increasing pulse width and strength, and the formations of large-size “nanopores” were mainly caused by pulse width.

### Effects of PEFs on cell fusion efficiency

**Fig 2:**
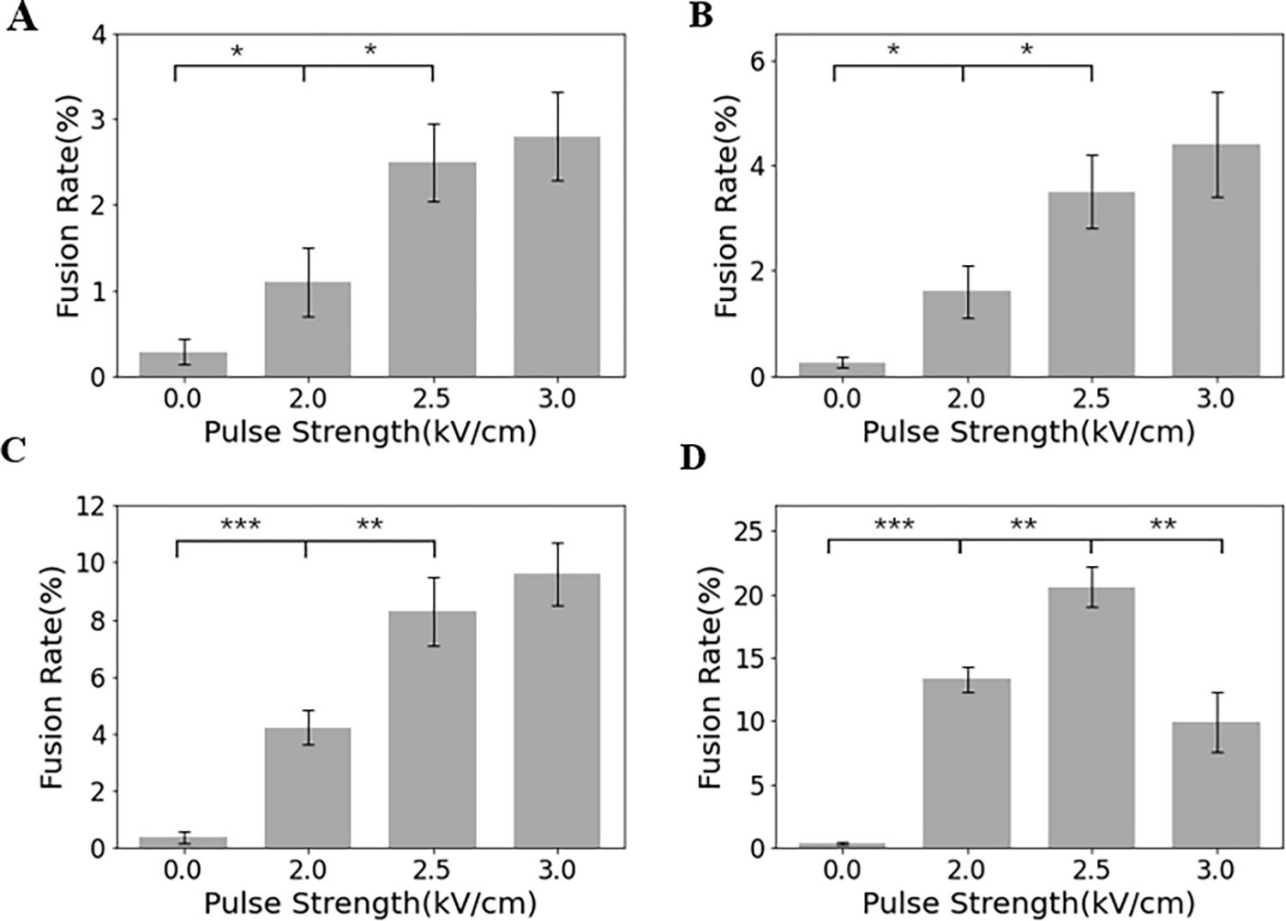
Cell fusion efficiencies with the pulse width of (A) 200ns, (B) 1000ns, (C) 10μs, and (D) 40μs. Fusion yields are means from five individual experiments; error bars show standard deviation. Asterisks *(P, 0.05), ** (P, 0.01) and *** (P, 0.001) mark significant difference between two groups.

Mouse SP2/0 myeloma cells were first incubated in hypoosmolar medium, then aligned through dielectrophoresis and exposed to pulses conditions with different widths (200 ns, 1000 ns, 10 μs or 40 μs) and strengths (0, 2.0, 2.5 or 3.0 kV/cm). Fig 2 shows that cell fusion yield increased with pulse width and strength. A significant increase can be observed in cell fusion efficiency when using longer pulses widths. The cell fusion efficiency in the width of 40 μs was almost twice that of the 10 μs, and five times that of the 1000 ns. These results suggested that larger pulse strength and longer width contributed to the higher cell fusion efficiencies. No statistically significant differences were observed between 2.5 and 3.0 kV/cm groups, implying that the fusion yields show a plateau.

Notably, when pulse widths were further increased to 60 μs, a noticeable drop in cell viability was observed, particularly under higher field strengths (3.0 kV/cm) (S3 Fig). This aligns with the observation in Fig 2D, where a drop in fusion yield occurred with the 40 μs and 3.0 kV/cm conditions, likely due to excessive cell electroporation. While moderate electroporation can promote cell fusion, exceeding a certain threshold in pulse width can cause irreversible damage to the cell membrane, leading to a loss of membrane integrity and functionality, ultimately resulting in cell death. This is consistent with previous observations, where a drop in fusion efficiency under the 40 μs and 3.0 kV/cm conditions was attributed to excessive electroporation. Previous evidences suggested that the irreversible electroporation leads to the leakage of cellular contents and permanent damage to membrane function [18]. Therefore, further increasing the pulse width significantly raises the rate of cell death, indicating a critical threshold for pulse width and strength beyond which the benefits of electroporation are outweighed by the detrimental effects of cell mortality.

### The correspondence between PEFs, electroporation and fusion efficiencies

To further explore the latent correspondence between PEFs, electroporation, and fusion efficiencies, PLS modeling was employed to establish a regression model for cell fusion rates. In this work, a total of 12 samples were randomly divided into 8 training samples and 4 test samples at a ratio of 2 : 1. In PLS modeling, the targeted variable was set as the cell fusion rates, and the variables were set to the electroporation results detected by PY and PI assays. From Table 1, it can be observed that the PLS model established on 8 training samples achieved good prediction performances.

**Table 1:** The PLS modeling results of electroporation rates.

Class	Name^†^	Electroporation rates by YP assay (%)	Electroporation rates by PI assay (%)	Experimental cell fusion rates (%)	Predicted cell fusion rates (%)
Training set	W_200ns_E_2.0_	78	8	1.10	2.62
	W_200ns_E_2.4_	83	12	2.50	3.48
	W_1000ns_E_0.0_	14	10	0.25	-1.11
	W_1000ns_E_2.4_	85	46	3.50	8.25
	W_10μs_E_0.0_	12	10	0.36	-1.24
	W_10μs_E_2.0_	81	53	4.20	8.96
	W_40μs_E_0.0_	10	8	0.31	-1.63
	W_40μs_E_2.4_	90	82	20.6	13.49
Test set	W_200ns_E_0.0_	13	10	0.28	-1.17
	W_1000ns_E_2.0_	82	38	1.60	6.97
	W_10μs_E_2.4_	86	62	8.30	10.50
	W_40μs_E_2.0_	87	76	13.3	12.48

^†^ Samples for PLS modeling. For example, W_200ns_E_2.0_ represents the sample with the pulse width of 200 ns and the electric field strength of 2.0 kV/cm. Electroporation rates with the electric field strength of 2.5 kV/cm were substituted for data with electric field strength of 2.4 kV/cm.

**Fig 3:**
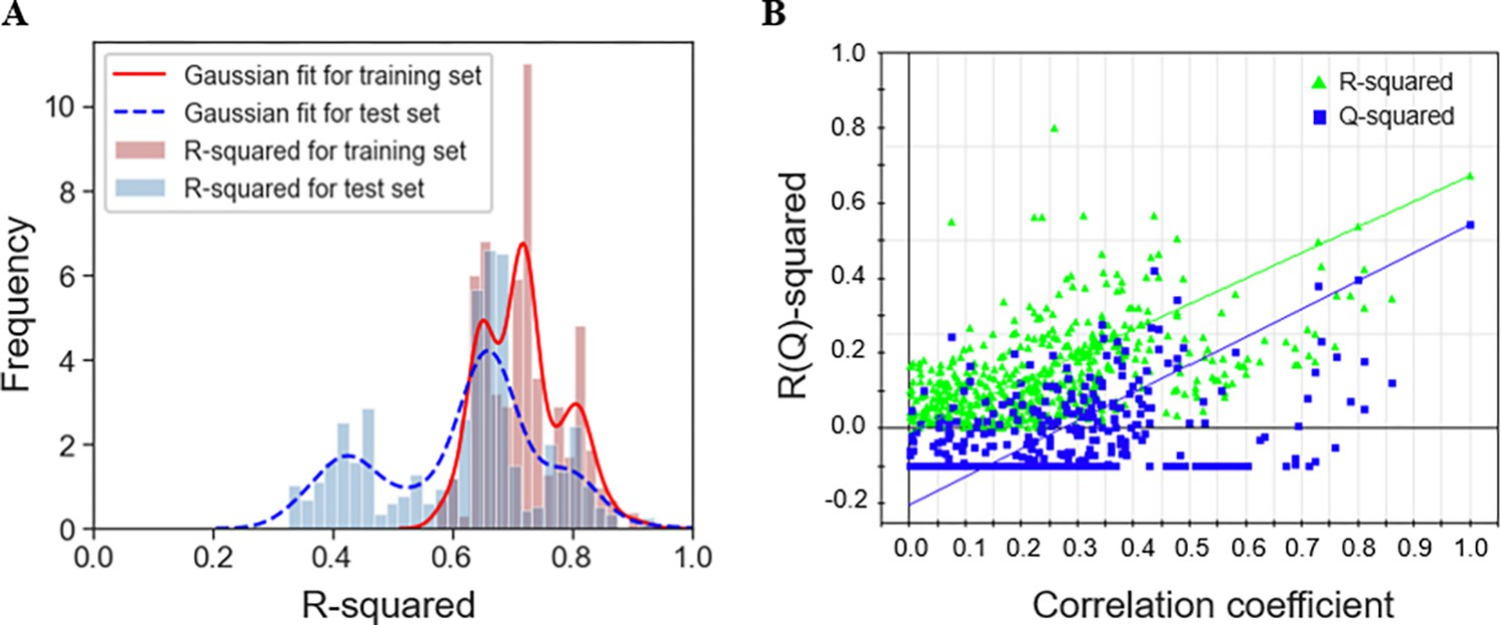
Results of PLS modeling validation. (A) *R*^*2*^ and *R*^*2*^_*test*_ distributions of 500-times repeated PLS modeling. (B) 500-times Y-random permutation test for the optimal PLS model.

To validate the robustness of PLS modeling, 500-times repeated PLS modeling and 500-times Y-random permutation test were performed. Fig 3A shows the frequency distribution of *R*^*2*^ and *R*^*2*^_*test*_ in 500-times repeated PLS modeling based on the randomly selected training and validation samples. The means of *R*^*2*^ and *R*^*2*^_*test*_ are 0.72 ± 0.07 and 0.62 ± 0.13, respectively. Besides, a 500-times Y-random permutation test was conducted for the optimal PLS model. It can be observed that the *R*^*2*^ and *Q*^*2*^ drop sharply along with the decreased correlation coefficients between the original and permuted Y, indicating that the high-quality PLS model is not caused by accident (Fig 3B).

**Fig 4:**
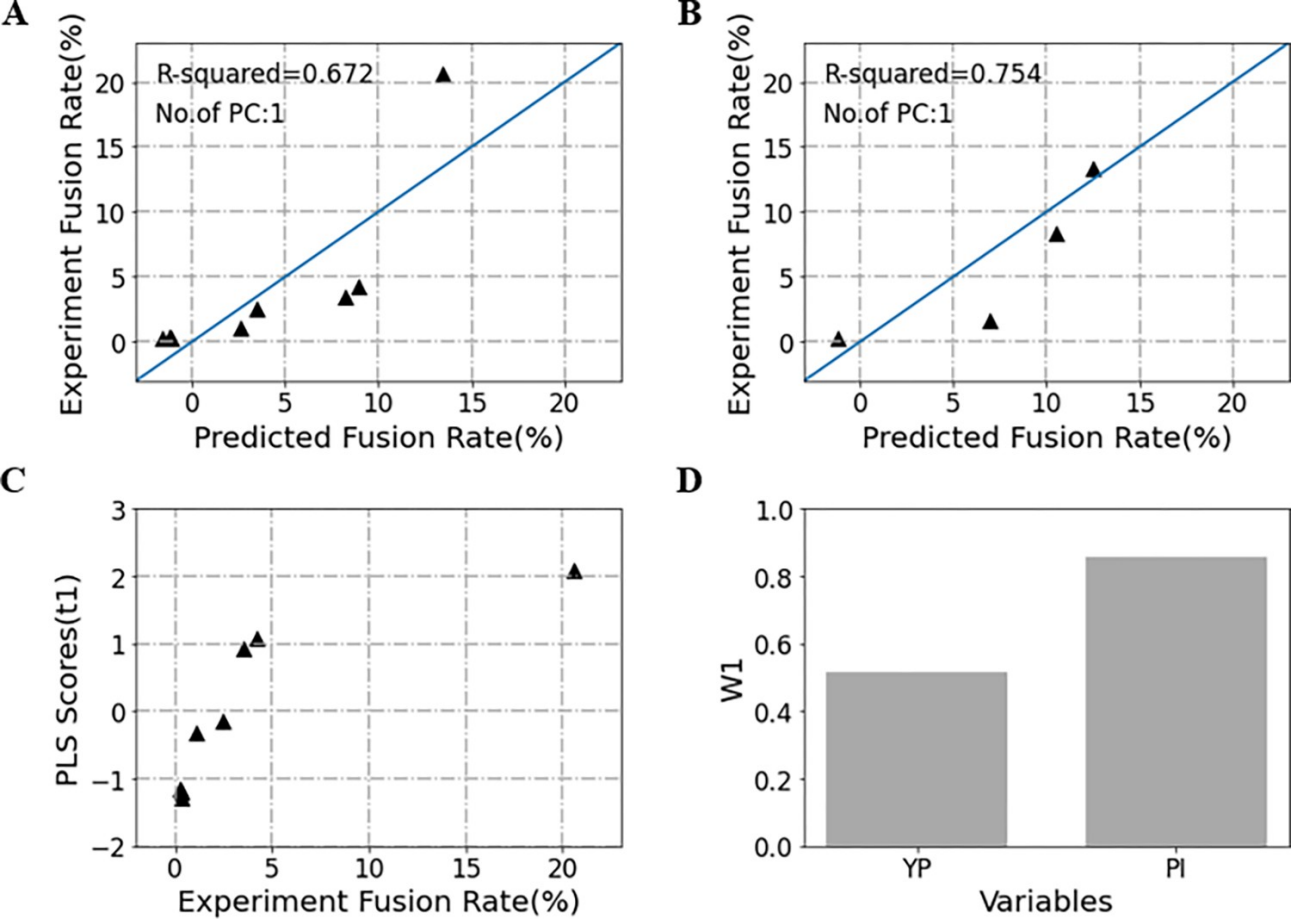
The PLS modeling results for the cell fusion efficiency of 12 samples. (A) The scatter plot of the experimental vs. predicted electroporation rates of the 8 training samples. (B) The scatter plot of the experimental vs. predicted electroporation rates of the 4 test samples. (C) The first principal component scores of the 8 training samples. (D) The weights of the independent variables PY and PI for the first principal component, of which the weights are 0.52 for YP and 0.86 for PI, respectively.

Fig 4A and 4B show the predicted vs. observed cell fusion rates of the 8 training and 4 test samples. All the samples are distributed very well along the regression lines from the origin. The PLS model with only one principal component (t1) shows excellent predictive performance, of which the R^2^ are 0.672 and 0.754, respectively. Fig 4C shows the first principal component (t1) scores of the 8 training samples. In the first principal component spaces, the experimental cell fusion rates increase gradually, which indicates that the first principal component (t1) can interpret most of the information well. The loading scatter plot of the PLS model shows that (Fig 4D), although both YP and PI variables contribute positively for the first principal component, the PI variables (weight = 0.86) make a greater contribution to the PLS modeling than the YP (weight = 0.52). That is to say, the formation of large-size “nanopores” is the most crucial key factor that improves the efficiencies of cell fusion.

### Molecular dynamics simulations

#### CG-based molecular simulations

**Fig 5:**
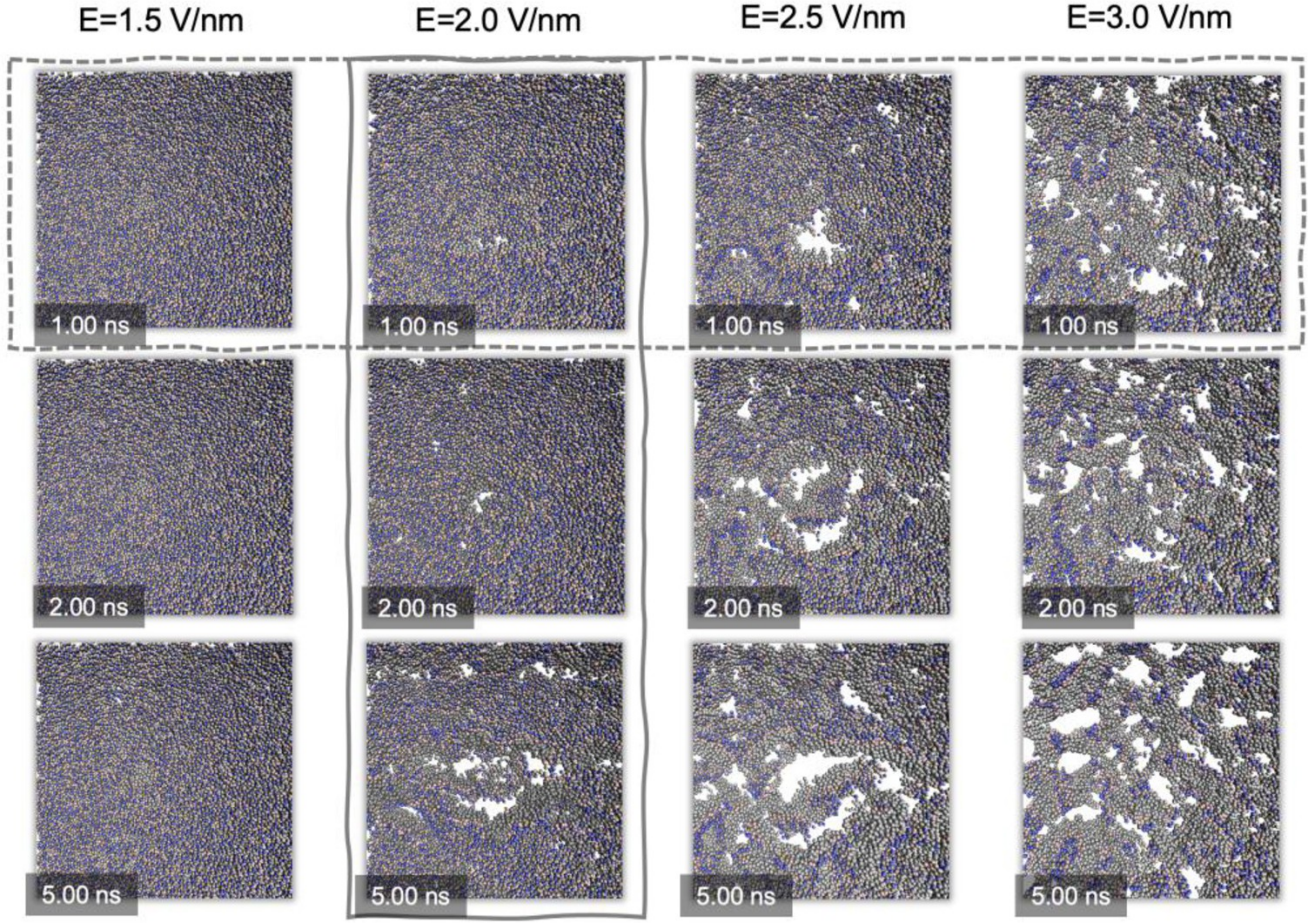
Morphological changes of CG-based DPPC bilayers during 10 ns MD simulations. The dotted box represents the morphological changes of DPPC bilayers with the same time point corresponding to different PEFs. The solid box represents the morphological changes of phospholipid bilayers over time under the same PEF conditions.

Herein, MD simulations based on DPPC phospholipid bilayers were first performed by the coarse-grained Martini force field. The static field was applied perpendicularly to DPPC bilayers. A serial of gradient pulse strength, i.e., 0.0, 0.5, 1.0, 1.5, 2.0, 2.5, 3.0 V/nm, were individually employed in 10-ns MD simulations to investigate the effects of pulse conditions on electroporation. Fig 5 shows the morphology evolutions of the DPPC membranes in different PEF conditions during electroporation. It can be seen that the phospholipid bilayers firstly expanded due to the increase of surface tension. However, the electroporation process would occur only in an electric field exceeding the pulse of 1.5 V/nm (Fig 5). In the cases of 2.0, 2.5, and 3.0 V/nm pulse, some visible defects emerged on the surface of phospholipid bilayers within 1 ns, and then gradually evolved into larger pores with the expansion of the membrane. Finally, the membrane expansion was complete within 5 ns and the pore sizes tended to be stable during the remaining simulation time.

Notably, although the number of pores increases slightly over time, it can be observed that the number of pores augments significantly when adopting a stronger pulse strength. Besides, the sizes of pores enlarge remarkably with the increase of pulse durations (Fig 5). In general, our findings on CG-based MD simulations confirmed the same trend with electroporation results, indicating that the pulse width is the critical element affecting the expansion of nanopores in the process of electroporation.

#### All-atom molecular simulations

**Fig 6:**
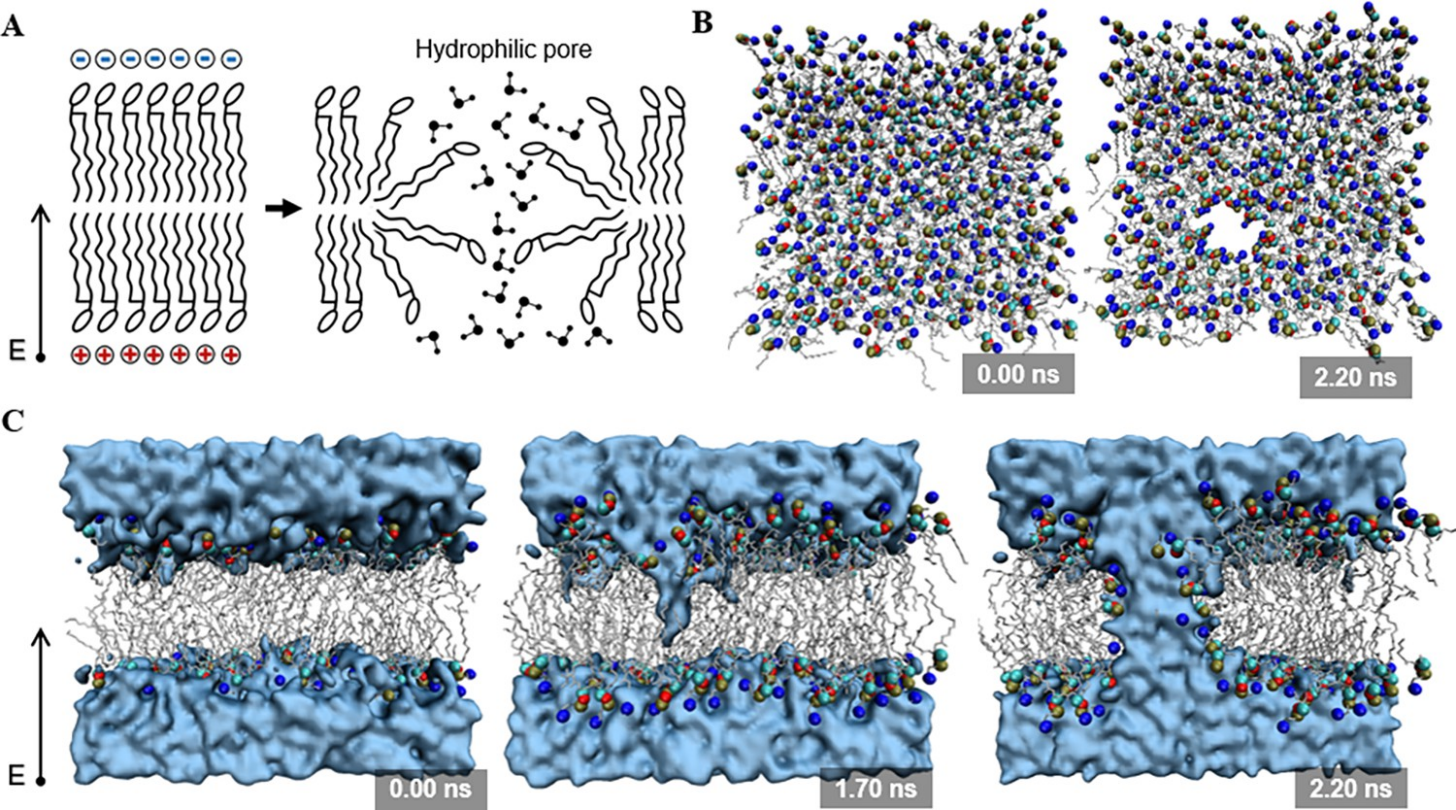
Electroporation events revealed by 10-ns all-atom MD simulation. (A) Conceptual scheme of theoretical electroporation mechanisms. (B) Top view of the pore creation in a membrane composed of 318 DPPC lipids at an electric field strength of E = 0.5 V/nm. (C) Snapshot of cross-sectional view during MD simulation of phospholipid bilayers electroporation (E = 0.5 V/nm). Alkyl chains are depicted as grey sticks. The head carbon atoms, phosphor atoms, choline groups, and lipid oxygen atoms are represented as cyan, tan, blue and red spheres, respectively. Water is shown in cerulean surface representation. The electric field (E) direction is represented as a black arrow.

The atomic details of pore creation were further explored by all-atom molecular simulations with a small unit comprised of 318 DPPC molecules. The mechanism and the key factor affecting pore creation were investigated by separately examining the pore morphology, pore volumes’ evolution and the potential distribution across the phospholipid bilayers during electroporation. In consideration of CG system is a simplified representation of the all-atom system and its analysis precision was relatively low, thus parameter range of electric field strength is different for all-atom and GC MD [40]. In this work, eleven gradient electric fields with different strengths (from 0 to 1.0 V/nm) were performed. Fig 6 shows the electroporation events revealed by all-atom MD simulations at the electric field strength of E = 0.5 V/nm. It can be seen that the pore formation is initiated by water fingers protruding at 1.7 ns, then the pore forms quickly within 0.5 ns. As expected for the theoretical electroporation event (Fig 6A), the water protruding is mainly caused by the rearrangement of the headgroup/glycerol region on both sides of the membrane. The water protruding first penetrates the hydrophobic core from the cathode monolayer (upper layer) of the membrane, and then expands toward the opposite membrane layer to form a water wire (Fig 6B and 6C).

**Fig 7:**
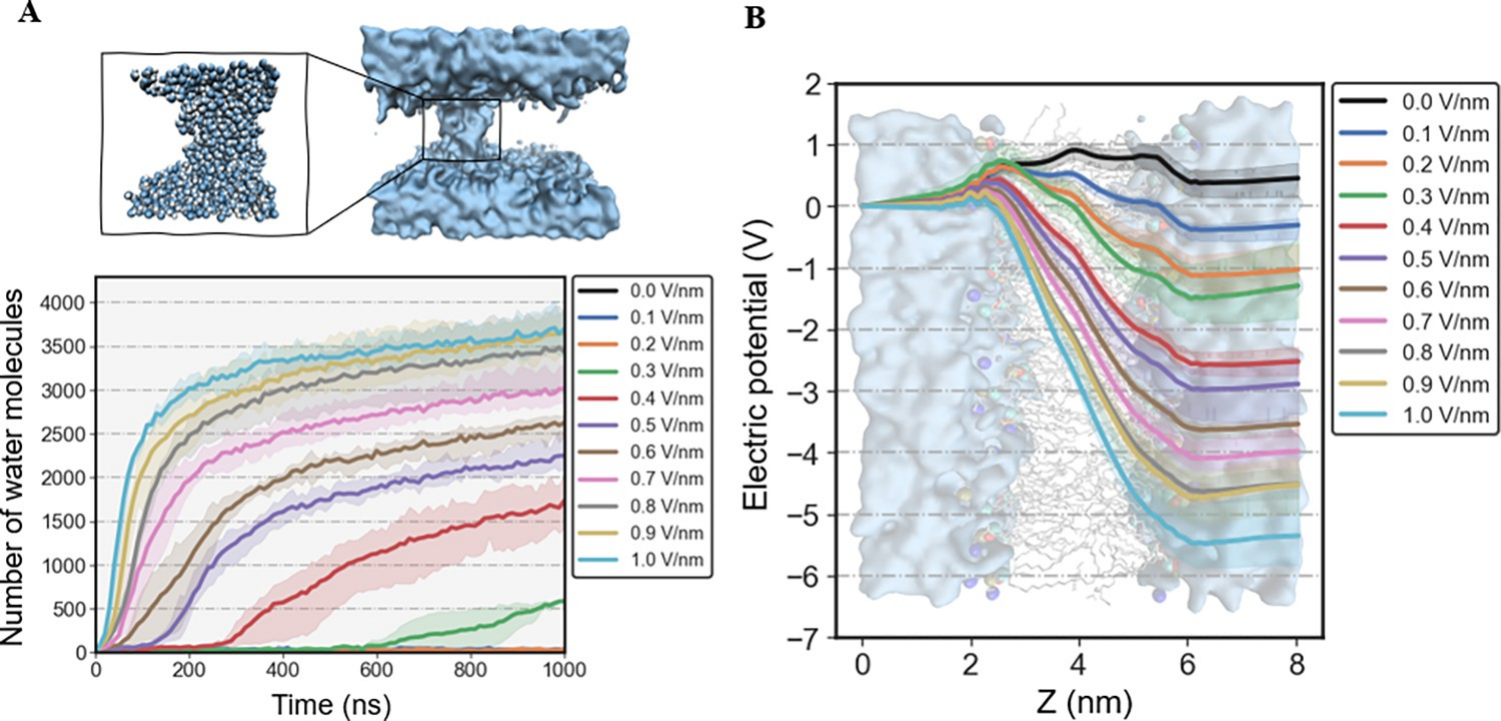
Pore volume and electrical potential distribution of DPPC bilayers in MD simulations. (A) Volume evolution of the hydrophilic pores during MD simulations. The volume of hydrophilic pores was determined by monitoring the amount of water between the two lipid layers. Water is shown in cerulean surface representation. The oxygen and hydrogen atoms of water molecules are represented as cerulean and white spheres, respectively. (B) Distribution of potential across the lipid membrane (along Z direction). The standard deviations were calculated from three-times independent MD simulations.

Similar to CG-based MD simulations, electroporation events occur only in an electric field exceeding the strength of threshold (E = 0.3 V/nm), and only one hydrophilic pore can be observed in each MD simulation due to the small size of membrane. As shown in Fig 7A, it can be observed that the pore volumes increase obviously over time. However, systems reached equilibrium quickly and the pore volume remained constant for the length of the simulations. In particular, increasing the strength of electric field can largely accelerate the expansion of pore volumes and reduce the time to reach equilibrium (Fig 7A).

Moreover, the potential in the direction perpendicular to the phospholipid bilayers was calculated to explore the electrical potential distributions of membrane in different electric field strengths. As shown in Fig 7B, it can be seen that the potential differences occur only at the interfaces between the upper/lower phospholipid membranes and the water phase. When the zero voltage is applied, the potential differences are negligible (< 0.1 V). While the potential differences significantly increase with increasing the strengths of electric field, indicating that the unbalanced degree of electric potential played a key role in the electroporation process. These results collectively imply that although increasing the pulse duration can significantly increase pore volumes, the upper limit for pore volumes in the electroporation process is mainly determined by the strength of electric field.

## Discussion

In this work, mouse SP2/0 myeloma cells were exposed to different PEF conditions to explore the influences of PEF parameters (i.e. pulse strengths and widths) on the electroporation and electrofusion of phospholipid bilayers. Experimental results and PLS regression analysis showed that large-size “nanopores” formations are mainly caused by pulse width. Our findings on CG-based and all-atom MD simulations further demonstrate the same trend with experiment results, which suggested that the number of pores increases with pulse strength, and the sizes of pores mainly increase with the pulse durations. The unbalanced degree of electric potential leads to a different pore size during the process of electroporation.

Our electroporation findings coincide with those of similar experiments conducted before. Nesin et al. [41] measured pore-sizes and volume in individually electroporated cells by using a time-lapse confocal imaging technique, and found that compared with the nanopores produced by 60-ns electric pulses, 600-ns pulses can generate a small fraction of larger pores. Similarly, Pakhomov et al. [42] explored the effects of nanosecond electric pulses on the pore size in electroporation, and proposed that the multiple pulses can increase the number of pores but not their size. These phenomena could be ascribable to the time inadequacy for pore expansions in the case of short pulse widths. By employing an amplitude close to the threshold of electroporation, Saulis et al. [43] investigated the size effect of the pores created by square-wave electric pulses with the duration of 100-μs and 2-ms. The results showed that the longer 2-ms duration pulse leads to larger pores than a short 100-μs duration pulse. In addition, several simulation studies carried out by the finite-element method also suggested that the high-strength pulse were mainly contributed to increasing the number of pores, and the pulse width is the most crucial factor for enlarging the nanopores in the process of electroporation [15,44]. It is noteworthy that previous studies have confirmed that the shape of electric pulses plays a significant role in electroporation. Depending on the intensity and duration of the pulses, the influence of other parameters, such as the number of pulses or their repetition frequency, will vary accordingly [45,46]. There is a tendency to move toward the shorter pulse duration range, which is a logical evolution of microsecond range electroporation.

Although comparatively few attempts have so far been made to investigate the correspondence between electroporation and electrofusion, another important assumption in this study is that the formation of large-size pores is the most crucial factor that affects the fusion rate of mouse myeloma cells. This finding supported the general consensus on electrofusion, that is to say, cell membranes fuse due to the spontaneous (or forced) bending of the pore edge when the pore radius exceeds the threshold value [47,48]. Thus, increasing the proportions of large-pores formation (or the number of PI-positive cells) might have contributed to a higher cell fusion efficiency in electrofusion experiments.

## Conclusion

Herein, influences of pulse strength and width on the electroporation and electrofusion of phospholipid bilayers were systematically investigated by using experiments combined with MD simulations. In general, its major conclusions and recommendations were as follows:

- Electroporation events occur only in an electric field exceeding the strength of threshold, and the pore formation and its sizes are determined by the unbalanced degree of electric potential between the upper and lower phospholipid membranes;
- PEFs can induce multiple pores on the membrane. The number of pores is mainly determined by pulse strength, while the sizes of pores were enlarged by increasing the pulse widths (or pulses duration);
- The formation of large-size pores is the most crucial factor that affects the cell fusion yield of mouse myeloma cells.

## Supporting information

S1 Fig The schematic diagram of the homemade instrument. (A) The cathodes of the pulse generator, power amplifier and oscilloscope were connected (black line). S1 and S2 were used to switch the sine waves and pulse waves, respectively. (B) Pulse waveforms with single-polarity pulse widths in the simulation.
(TIF)

S2 Fig System preparations of MD simulations. (A) Schematic diagram of CG and all-atom models in the water box. The representative models were intercepted along the X-axis from bilayers models. (B) Correspondence relationships between the CG and all-atom models for DPPC molecules. NC3: Choline group; PO4: Phosphate group; GL: Glycerol group; C1-4: Alkyl chains. (C) Snapshot in all-atom MD simulation of phospholipid bilayers electroporation (E = 0.7 V/nm) under the conditions of non-constraint and with harmonically constrained by a force of 100 kcal/mol·Å^2^. (d) The range of harmonic constraints in all-atom model is colored green. The glycerol Cα-atoms and DPPC backbone are represented as spheres and lines, respectively.
(TIF)

S3 Fig Cell staining experiment. (A) Cell staining experiment of SP2/0 cells and lymphocytes (2.5 kV/cm, 40 μs). Cells formed pearl chain alignment upon application of sine voltage (left). The Control group was captured 25 min after applying sine voltage. Applying a sine voltage alone without a pulsed electric field (medium). The Experimental group of 2.5 kV/cm was captured 25 min. Red fluorescence indicates dead cells (right); Four hybrid cells were found (I, II, III and IV). Red fluorescence indicates dead cells. (B) Changes of cell fusion rate and mortality rate with different pulse width.
(TIF)
