# Supplementary figures and images for: Influences of pulsed electric field parameters on cell electroporation and electrofusion events: Comprehensive understanding by experiments and molecular dynamics simulations
Source: PLoS One. 2025 Jan 22;20(1):e0306945. doi: 10.1371/journal.pone.0306945 (PMC11753653; doi:10.1371/journal.pone.0306945)

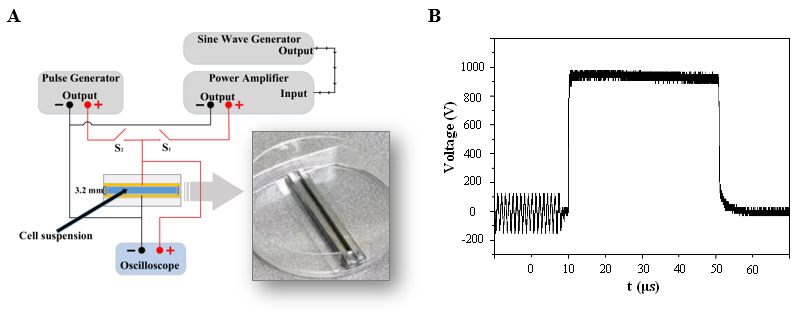

Supplement: S1 Fig — (A) The cathodes of the pulse generator, power amplifier and oscilloscope were connected (black line). S1 and S2 were used to switch the sine waves and pulse waves, respectively. (B) Pulse waveforms with single-polarity pulse widths in the simulation. (TIF) [file pone.0306945.s001.tif]

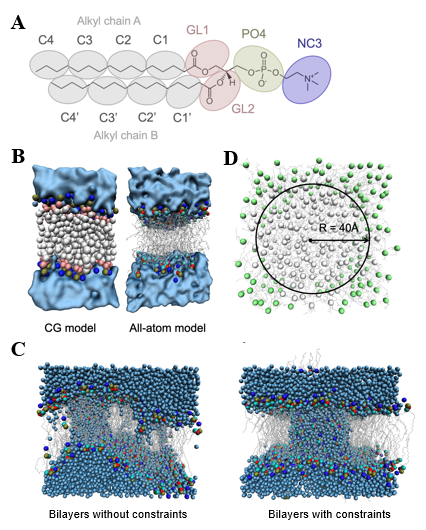

Supplement: S2 Fig — (A) Schematic diagram of CG and all-atom models in the water box. The representative models were intercepted along the X-axis from bilayers models. (B) Correspondence relationships between the CG and all-atom models for DPPC molecules. NC3: Choline group; PO4: Phosphate group; GL: Glycerol group; C1-4: Alkyl chains. (C) Snapshot in all-atom MD simulation of phospholipid bilayers electroporation (E = 0.7 V/nm) under the conditions of non-constraint and with harmonically constrained by a force of 100 kcal/mol·Å2. (d) The range of harmonic constraints in all-atom model is colored green. The glycerol Cα-atoms and DPPC backbone are represented as spheres and lines, respectively. (TIF) [file pone.0306945.s002.tif]

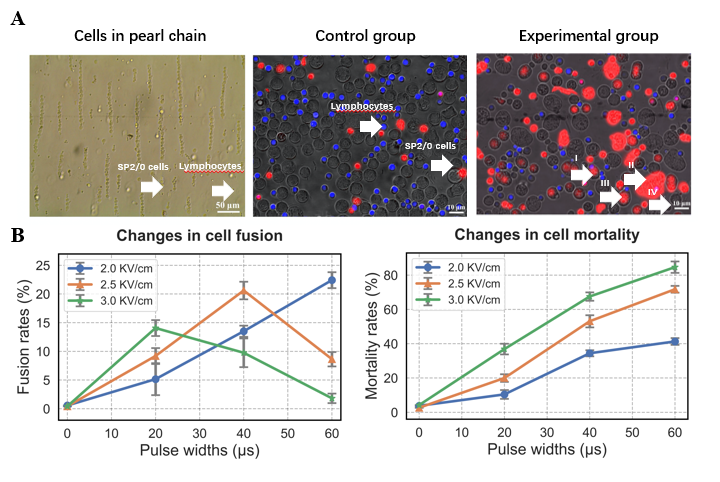

Supplement: S3 Fig — (A) Cell staining experiment of SP2/0 cells and lymphocytes (2.5 kV/cm, 40 μs). Cells formed pearl chain alignment upon application of sine voltage (left). The Control group was captured 25 min after applying sine voltage. Applying a sine voltage alone without a pulsed electric field (medium). The Experimental group of 2.5 kV/cm was captured 25 min. Red fluorescence indicates dead cells (right); Four hybrid cells were found (I, II, III and IV). Red fluorescence indicates dead cells. (B) Changes of cell fusion rate and mortality rate with different pulse width. (TIF) [file pone.0306945.s003.tif]
